# Tree shrew as a new animal model to study the pathogenesis of avian influenza (H9N2) virus infection

**DOI:** 10.1038/s41426-018-0167-1

**Published:** 2018-10-10

**Authors:** Runfeng Li, Bing Yuan, Xueshan Xia, Sheng Zhang, Qiuling Du, Chunguang Yang, Na Li, Jin Zhao, Yunhui Zhang, Rongping Zhang, Yue Feng, Jianlin Jiao, Malik Peiris, Nanshan Zhong, Chris Ka Pun Mok, Zifeng Yang

**Affiliations:** 1 State Key Laboratory of Respiratory Disease, National Clinical Research Center for Respiratory Disease, Guangzhou Institute of Respiratory Health, the First Affiliated Hospital of Guangzhou Medical University, Guangzhou, Guangdong P.R. China; 2 Department of Respiration, First People’s Hospital of Yunnan Province, Kunming, Yunnan P.R. China; 3 Faculty of Life Science and Technology, Kunming University of Science and Technology, Kunming, P.R. China; 4 School of Pharmaceutical Science & Yunnan Key Laboratory of Pharmacology for Natural Products, Kunming Medical University, Kunming, Yunnan P.R. China; 5 Technology Transfer Center, Kunming Medical University, Kunming, Yunnan P.R. China; 6 The HKU–Pasteur Research Pole, School of Public Health, Li Ka Shing Faculty of Medicine, The University of Hong Kong, Hong Kong, China

## Abstract

Outbreaks of avian influenza virus continue to pose threats to human health. Animal models such as the mouse, ferret, and macaque are used to understand the pathogenesis of avian influenza virus infection in humans. We previously reported that the tree shrew (*Tupaia belangeri*, family Tupaiidae), which is regarded as a “low-level primate”, has α2,3- and α2,6-linked sialic acid receptor distributions similar to those of humans and is potentially a useful mammalian model for studying mild human influenza (H1N1) virus infection. In this study, we used the tree shrew experimental model to investigate the pathogenesis of avian influenza A (H9N2) virus infection and the effect of the E627K mutation in the *PB2* gene, an adaptation to mammalian hosts. Evidence of disease, virus titers in the upper and lower respiratory tract, histopathology and induction of proinflammatory cytokines are described. We also established ex vivo culture models of tree shrew respiratory tissues to study the tropism and replication of the H9N2 virus. Our results demonstrated that the tree shrew is a viable new in vivo experimental model for avian influenza research that provides results comparable to those observed in ferrets. The disease spectrum and pathogenesis in tree shrews correlate well with what is observed in humans.

## Introduction

Influenza A (H9N2) virus is the most widespread subtype of avian influenza found in poultry in Asia. In addition to the economic impact on the poultry industry, interspecies transmission of H9N2 viruses from poultry to humans has occurred repeatedly^1–4^, and the virus poses a potential pandemic threat. Moreover, reassortment between H9N2 and other avian viruses has generated new zoonotic subtypes, such as H5N1, H7N9, and H10N8, which have caused lethal zoonotic disease^5–7^. The H9N2 viruses that are circulating in poultry in China have evolved into two major phylogenetic lineages: the H9N2/G1-like lineage, represented by A/Quail/Hong Kong/G1/97 (H9N2/G1), and the H9N2/Y280-like lineage, represented by A/Duck/Hong Kong/Y280/97 (H9N2/Y280)^8^. Although H9N2 virus can cause severe disease in immunocompromised patients, most human infections in immunocompetent persons are associated only with mild respiratory disease^2–4^. In vivo studies have shown that a mutation from glutamic acid (E) to lysine (K) at amino acid residue 627 of the *PB2* gene is an important adaptation marker of avian influenza viruses that contributes to pathogenesis in mammalian hosts^9–11^.

Mouse and ferret have been commonly used to study the pathogenesis of H9N2 viruses in mammalian hosts. However, the distribution of sialic acids (SA)—which act as receptors for influenza viruses—differs notably between mice and humans. Many influenza viruses that infect humans do not infect mice without prior adaptation. Meanwhile, H9N2 infection in mice causes severe inflammation in the lower part of the lung, which is not what is usually observed in humans^12–14^. On the other hand, H9N2 viruses cause mild respiratory symptoms in the ferret, approximating human infection^15,16^. However, the use of ferrets is limited by the relatively high cost and by the requirement for specially designed facilities and husbandry. Cynomolgus macaques have been used as experimental models for influenza^17,18^. However, the SA receptor distribution in macaques does not parallel what is found in humans, and the facilities needed for macaques are not widely available.

The tree shrew (*Tupaia belangeri*, family Tupaiidae), indigenous to South Asia, Southeast Asia, and Southwest China, is classified as a separate taxonomic group of mammals that diverged from the primate order approximately 85 million years ago but still be considered as a “low-level primate” based on their close relationship^19^. Tree shrew is phylogenetically more closely related to humans than ferret and mice. We have previously shown that the tree shrew supports the replication of human influenza viruses without prior adaptation by serial passages and that the resulting infections bring mild disease symptoms resembling those in humans^20^. Importantly, we showed that the distribution of SA receptors in the respiratory tract of tree shrews is similar to that of humans. Recently, the genome of the tree shrew has been fully sequenced, which will help to establish research tools for use in a tree shrew model^21^. In this study, we investigated whether the tree shrew is susceptible to infection with avian influenza (H9N2) virus, and we evaluated the virologic and immunological features of the H9N2-infected tree shrews through in vivo and ex vivo models.

## Results

### Infection with H9N2 viruses in tree shrews and ferrets showed comparable pathogenicity

**Fig. 1:**
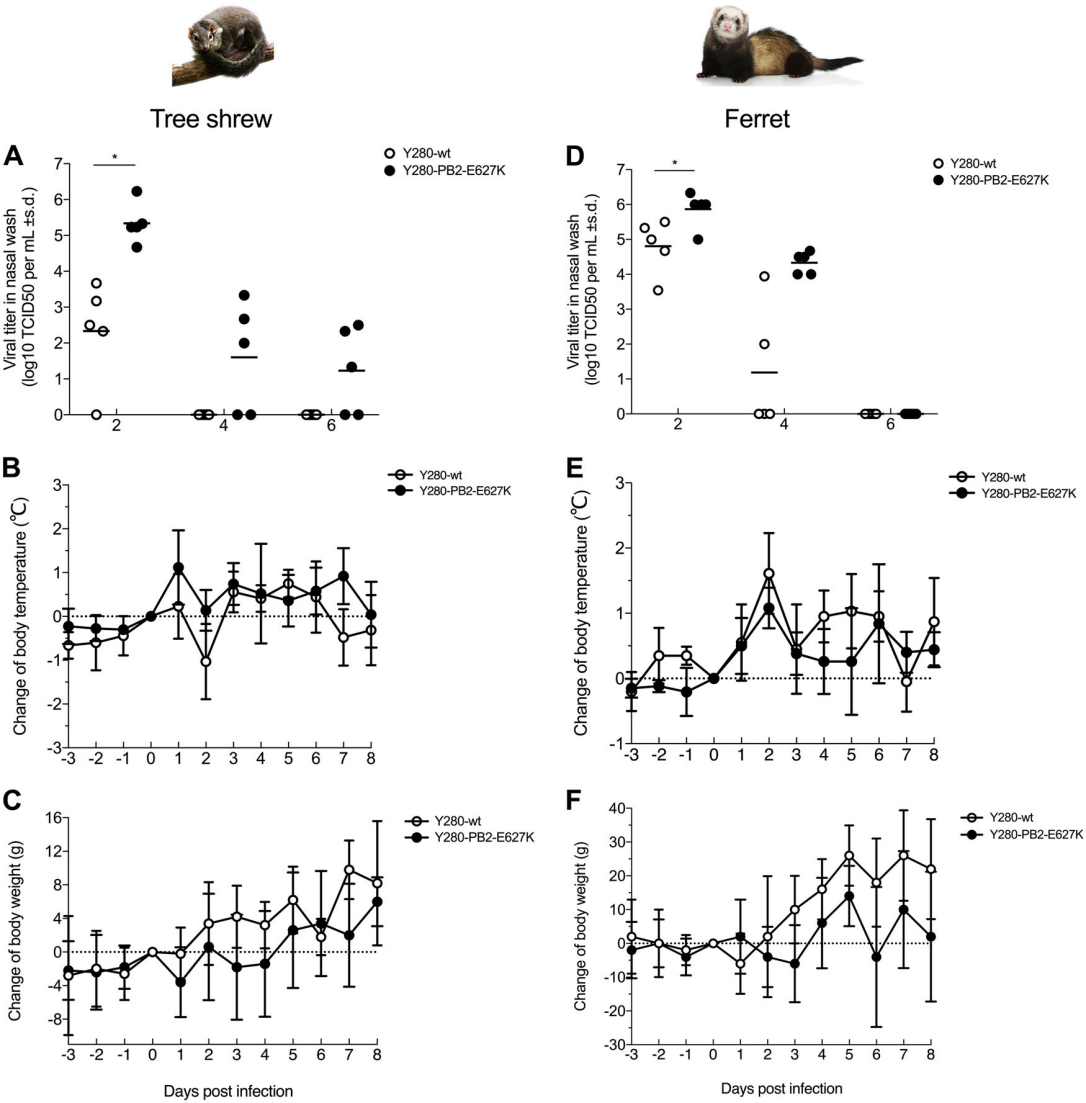
Susceptibility to H9N2 virus infection in tree shrews and ferrets. Viral titers in nasal wash (**a**, **d**) and changes in body temperature (**b**, **e**) and body weight (**c**, **f**) were determined in tree shrews and ferrets (*n* = 5 per group) infected with 10^6^ TCID_50_ of two different H9N2 viruses. Asterisks indicate a significant difference between the Y280-wt and Y280-PB2-E627K viruses. *p < 0.05

To determine whether tree shrews are susceptible to infection with avian H9N2 virus without prior virus adaptation and to compare the pathogenicity with that in ferrets, we infected tree shrews and ferrets with 10^6^ TCID_50_ of the H9N2 Y280-wt virus. To investigate the role of the mammalian adaptation mutation PB2-E627K, a Y280 virus (PB2-E627K) by reverse genetics and tested in parallel with the Y280-wt virus (Fig. 1). With the exception of one tree shrew infected with Y280-wt virus, virus shedding was found in the nasal wash of all other tree shrews and ferrets 2 days after infection (Fig. 1a, d). The PB2-E627K mutant replicated more quickly than the wild-type isotype on day 2 in both tree shrews and ferrets. In tree shrews, the virus could be detected until day 6 in the group challenged by the PB2-E627K mutant, while the wild-type virus was not found in any of the tree shrews on day 4. Shedding of both viruses was found until day 4 in the ferrets. Seroconversion to the challenged H9N2 subtype was found in all animals by hemagglutination inhibition (HI) assays, with antibody titers ranging from 80 to 640 in tree shrews and from 80 to 2560 in ferrets (Table 1). No animal died during the 14-day observation period.

**Table 1:** Susceptibility of tree shrews and ferrets to infection with H9N2 viruses

Virus strain	Peak titer in nasal wash ± SD (day)^a^		(+) viral titer in nasal wash^b^		Seroconversion (range of the HI titer)^c^	
	Tree shrew	Ferret	Tree shrew	Ferret	Tree shrew	Ferret
Y280-wt	2.34 ± 1.41 (2)	4.81 ± 0.77(2)	4/5	5/5	5/5 (336, 80–640)	5/5(720, 80–1280)
Y280-PB2-E627K	5.34 ± 0.56 (2)	5.87 ± 0.50(2)	5/5	5/5	5/5 (512, 320–640)	5/5 (2030, 1280–2560)

^a^Peak nasal wash titers are expressed as the mean log_10_TCID_50_/mL ± SD

^b^The lower limit of detection was 10^1.5^ TCID_50_/mL on day 2 postinfection

^c^Serum samples were collected on day 21 postinfection, and the HI titers were determined by using chicken RBCs

We next compared changes in body temperature and body weight between tree shrews and ferrets following H9N2 virus infection. Although there was no statistically significant difference between the two groups, some tree shrews infected with the Y280-PB2-E627K viruses showed an increase in body temperature and an increased loss of body weight at 1-day postinfection compared to the mean value of those infected with the Y280-wt virus (Fig. 1b, c). Mock-infected tree shrews showed no significant change in body temperature, but their body weight increased (Supp Fig. 2a, b). In the ferret model, both the Y280-wt and Y280-PB2-E627K viruses brought an obvious increase in body temperature and weight loss during the first few days of infection. Ferret infected with the wild-type virus regained weight more quickly than those infected with the PB2-E627K mutant (Fig. 1e, f).

### H9N2 virus replication in the nasal turbinates and lungs of tree shrews

**Fig. 2:**
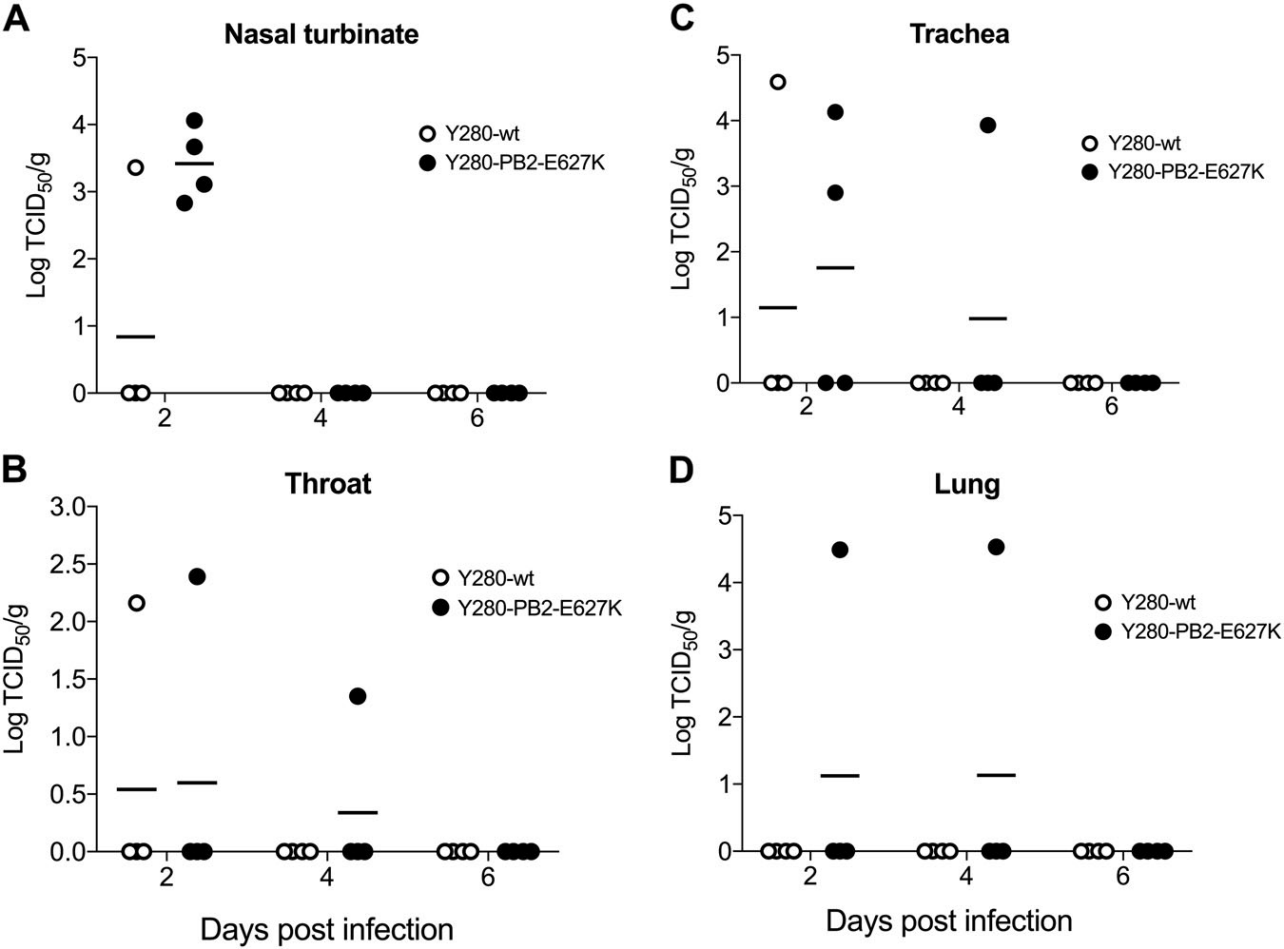
Viral titers from different tissues of the respiratory tract in tree shrews infected with H9N2 viruses. Tree shrews (*n* = 4 per group) were infected with 10^6^ TCID_50_ of Y280-wt or Y280-PB2-E627K virus. Nasal turbinate (**a**), throat (**b**), trachea (**c**), and lung (**d**) tissues were collected at 2, 4, and 6 dpi. The supernatant of the tissue homogenate was subjected to a TCID_50_ assay using MDCK cells. The dots represent the viral titer (log_10_TCID_50_ per gram of tissue) of individual animals; the lines indicate the mean value in each group

We next investigated the replication of H9N2 viruses in different tissues of the respiratory tract in infected tree shrews. Nasal turbinate, throat, trachea, and lung tissues were collected at 2, 4, and 6 days postinfection (dpi), and the viral titer from the supernatant of the homogenized tissues was determined by TCID_50_ assays. Consistent with the results from the nasal wash, Y280-PB2-E627K virus showed higher viral replication than Y280-wt in nasal turbinate at 2 dpi, but neither viruses showed any replication at 4 or 6 dpi (Fig. 2a). Replication in the throat and trachea could be found only in some tree shrews infected with either Y280-wt or Y280-PB2-E627K virus (Fig. 2b, c). Virus replication in the lungs was found only at 2 and 4 dpi in the two tree shrews infected with Y280-PB2-E627K virus (Fig. 2d).

### Histopathology in the respiratory epithelium of tree shrews after infection with H9N2 virus

**Fig. 3:**
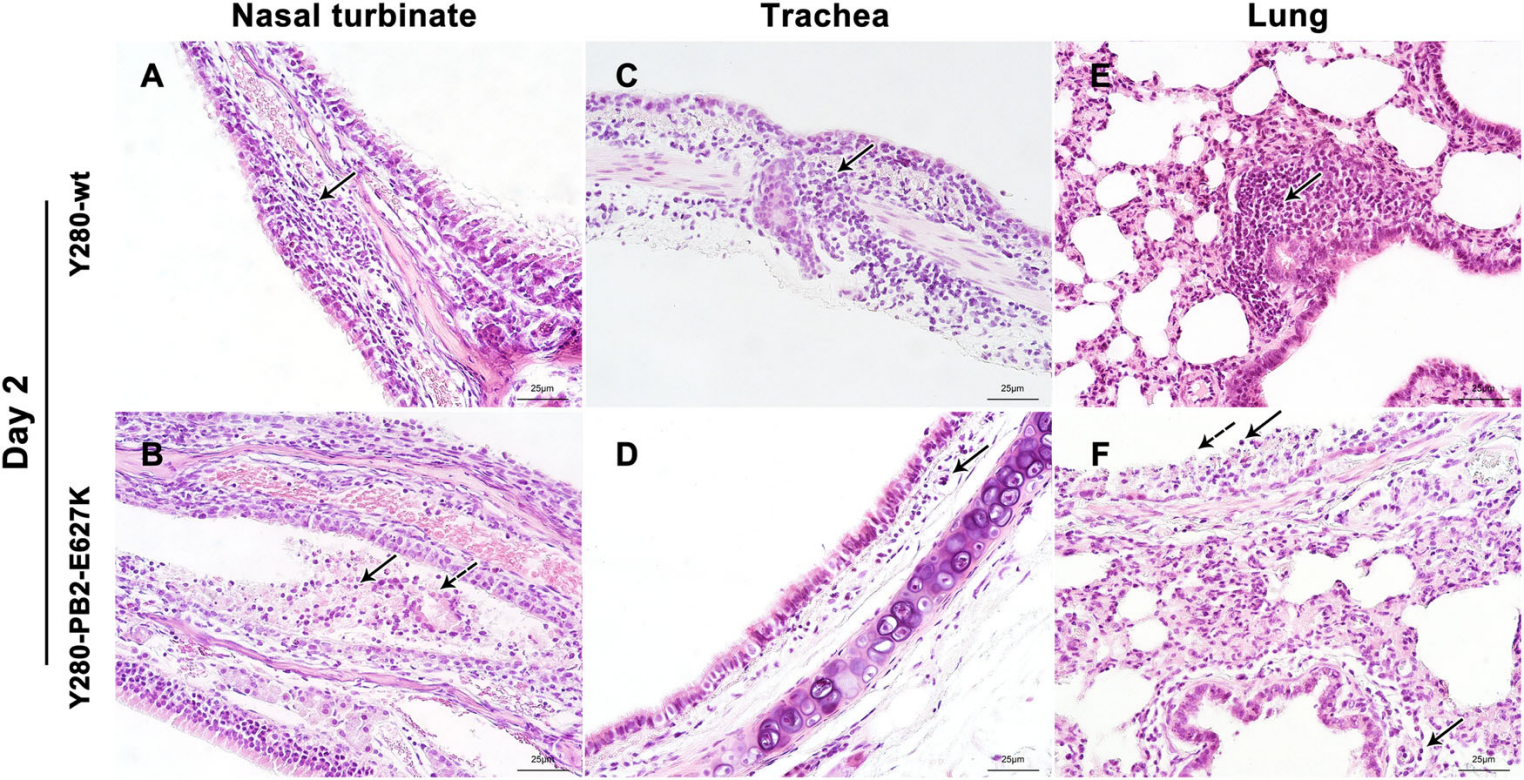
Histopathology of the respiratory epithelium at 2 dpi in tree shrews infected with H9N2 viruses. Tree shrews (*n* = 4 per group) were infected with 10^6^ TCID_50_ of one of two H9N2 viruses (Y280-wt or Y280-PB2-E627K). Nasal turbinate (**a**, **b**), trachea (**c**, **d**), and lung (**e**, **f**) tissues were collected at 2 dpi, processed into paraffin sections and stained with H&E. Black arrows indicate infiltration of inflammatory cells, and dotted arrows indicate necrotic and sloughed epithelial cells. The images are shown at ×400 magnification

**Fig. 4:**
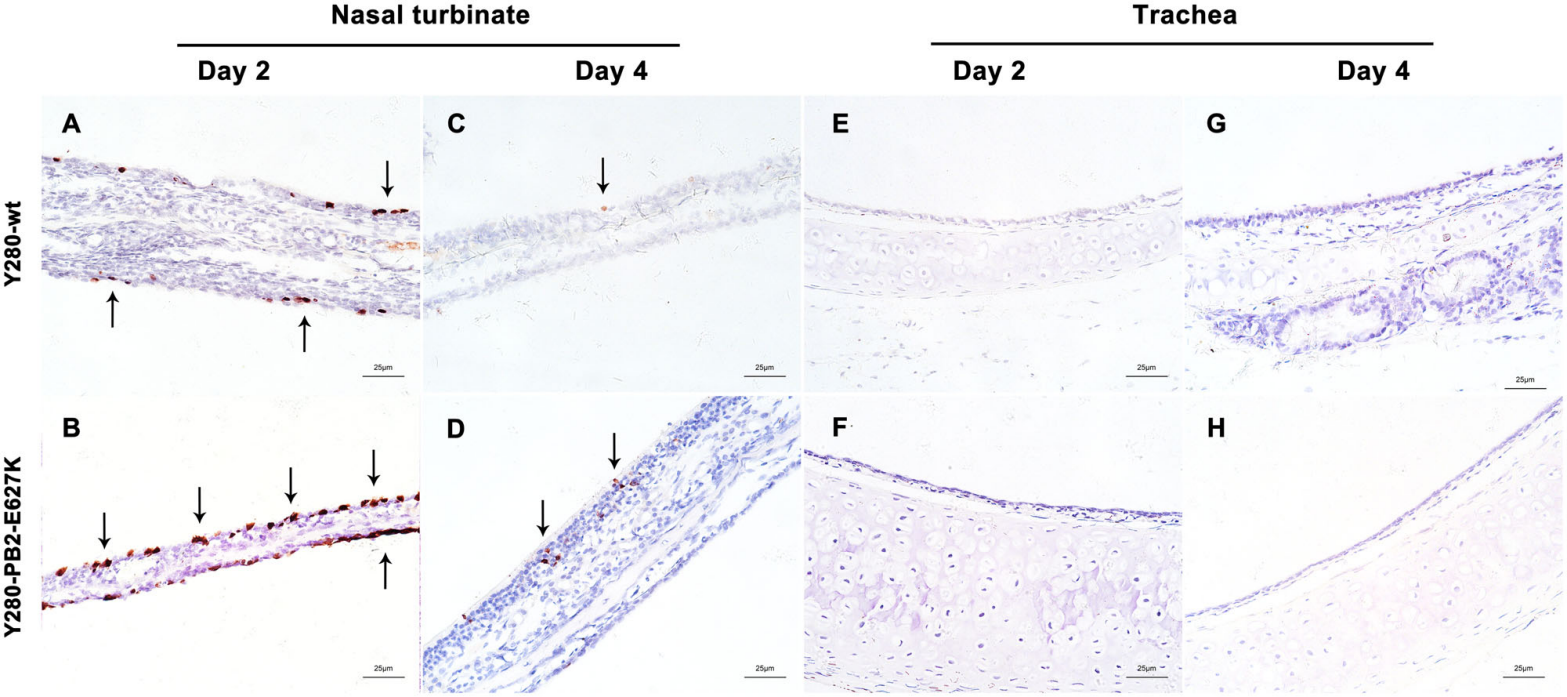
Immunohistochemical staining of respiratory epithelium from tree shrews infected with H9N2 viruses. Tree shrews (*n* = 4 per group) were inoculated with 10^6^ TCID_50_ of H9N2 viruses (Y280-wt or Y280-PB2-E627K). Nasal turbinate (**a**–**d**) and trachea (**e**–**h**) tissue were collected at 2 and 4 dpi, processed into paraffin sections and stained for the presence of influenza virus antigen by IHC. The images are shown at ×400 magnification

Inflammatory cells (lymphocytes and neutrophils) and focal edema on the submucosal layer of nasal turbinate were found in the tree shrews infected with Y280-wt virus at 2 dpi (Fig. 3a). The tissues isolated from the Y280-PB2-E627K-infected group showed an increased severity of lesions on the nasal turbinate, which were characterized by the identification of necrotic and sloughed epithelial cells, increased lymphocytic infiltration in the submucosal/intramucosal layers and vascular dilatation (Fig. 3b). Increased expression of viral antigens was detected in the epithelium of nasal turbinate tissues from the Y280-PB2-E627K-infected group compared to those infected with the Y280-wt virus (Fig. 4a–d). At 4 dpi, the severity of the lesions on the nasal turbinate was significantly improved in both groups (Supp Fig. 1a, b). The tracheal epithelium of both groups showed mild lymphocytic inflammation of the submucosal/intramucosal layers (Fig. 3c, d and Supp Fig. 1C, D). No virus antigen was identified in the trachea obtained from either group (Fig. 4e–h). While there was no evidence of virus replication in the lung, the peribronchiolar epithelium of the Y280-wt-infected tree shrews still showed mild infiltration of macrophages and thickened alveolar walls at 2 and 4 dpi (Fig. 3e and Sup Fig. 1E). Virus replication was found in the lung tissues of two tree shrews infected with the Y280-PB2-E627K virus, one at 2 dpi and one at 4 dpi (Fig. 2d). Interestingly, the lung tissues isolated from the Y280-PB2-E627K-infected group, including those with and those without positive detection of virus replication, all showed increased severity of lesions, including apparent necrotic and sloughed cells as well as inclusion bodies in the mucosal layer of bronchiolar epithelium, compared to the tree shrews infected with the Y280-wt virus (Fig. 3f and Supp Fig. 4F). The histopathology and immunohistochemical staining of mock-infected tree shrews are shown as controls (Supp Fig. 2C, D).

### Expression profile of cytokine mRNAs in tree shrews infected with the H9N2 viruses compared with mock-infected controls

**Fig. 5:**
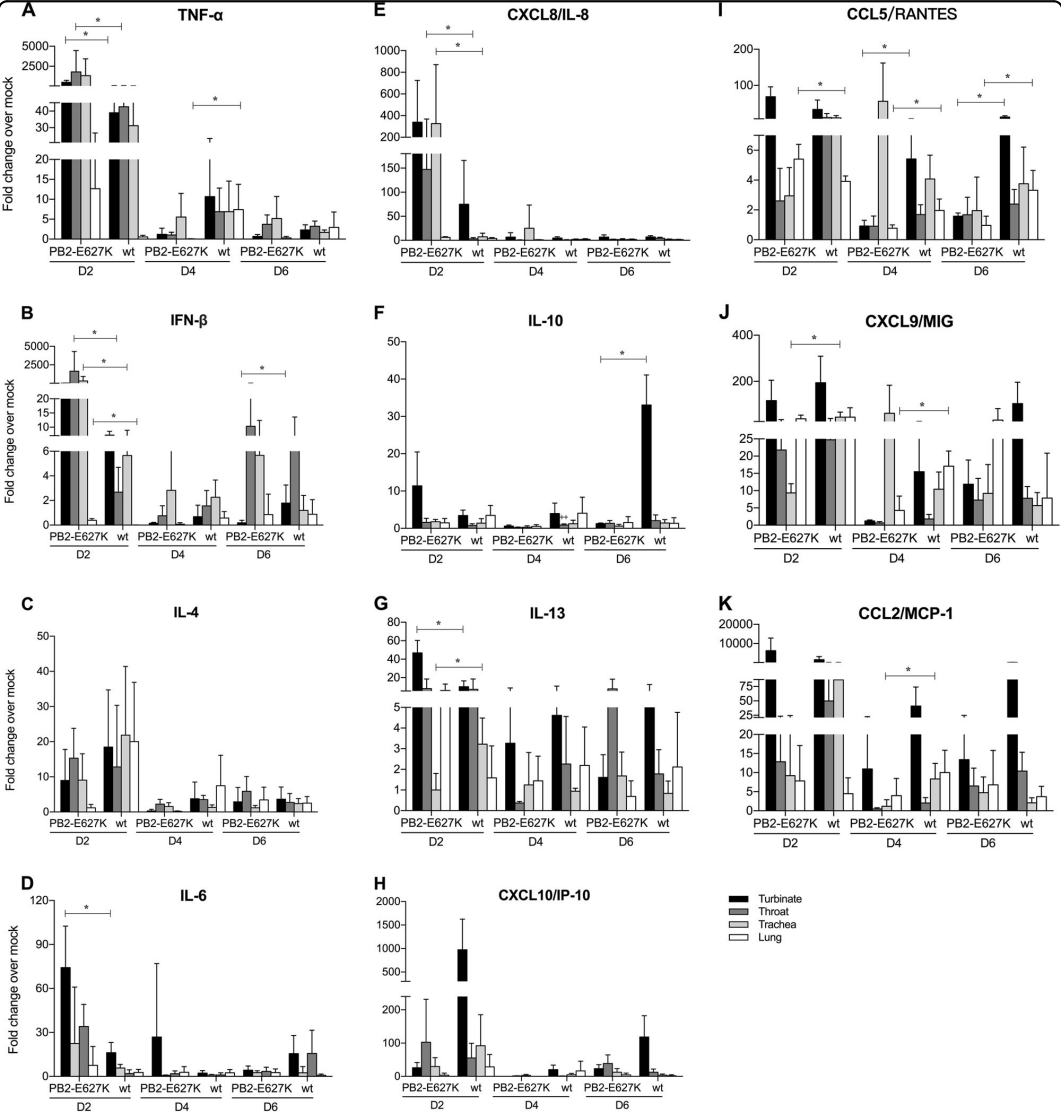
Cytokine mRNA expression in different tissues of the tree shrew respiratory tract. The mRNA expression levels of *TNF-α* (**a**), *IFN-β* (**b**), *IL-4* (**c**), *IL-6* (**d**), *CXCL8* (**e**), *IL-10* (**f**), *IL-13* (**g**), *CXCL10* (**h**), *CCL5* (**i**), *CXCL9* (**j**), and *CCL2* (**k**) were determined by quantitative PCR in the nasal turbinate, throat, trachea, and lung tissue of tree shrews (*n* = 4 per time point) that were infected with H9N2 viruses (Y280-wt or Y280-PB2-E627K). The expression of the target genes was standardized to the mRNA expression of the *GAPDH* gene and the expression in mock-infected tree shrews. *p < 0.05

The mRNA expression profiles of selected cytokines, including *tumor necrosis factor α (TNF-α), interferon β (IFN-β), IL-4, IL-6, CXCL8 (IL-8), IL-10, IL-13, CXCL10 (IP-10), CCL5 (RANTES), CXCL9 (MIG),* and *CCL2 (MCP-1)*, were measured from nasal turbinate, throat, trachea, and lung tissues obtained from tree shrews infected with the H9N2 viruses. The results are expressed as fold change compared to mock-infected tissue. In general, tree shrews infected with either wild-type or PB2 mutant H9N2 virus showed higher induction of cytokines in the examined tissues than the uninfected controls. In general, the expression of all cytokines peaked at 2 dpi (Fig. 5). At this time point, we found that the mRNA levels of *TNF-α, IFN-β, IL-8, IL-13,* and *IL-6* in the upper respiratory tract (nasal turbinate or throat) and *IL-8, RANTES*, and *IFN-β* in the lower respiratory tract (trachea or lung) were significantly higher in the tree shrews infected with Y280-PB2-E627K than in those infected with Y280-wt virus. On the other hand, Y280-wt virus triggered significantly higher mRNA expression of *MIG* (2 dpi, trachea; 4 dpi, lung), *IL-13* (2 dpi, trachea), *MCP-1* (4 dpi, trachea), and *IL-10* (6 dpi, nasal turbinate) than Y280-PB2-E627K virus.

### Virus replication and cytokine mRNA expression of the tree shrew ex vivo cultures after H9N2 virus infection

**Fig. 6:**
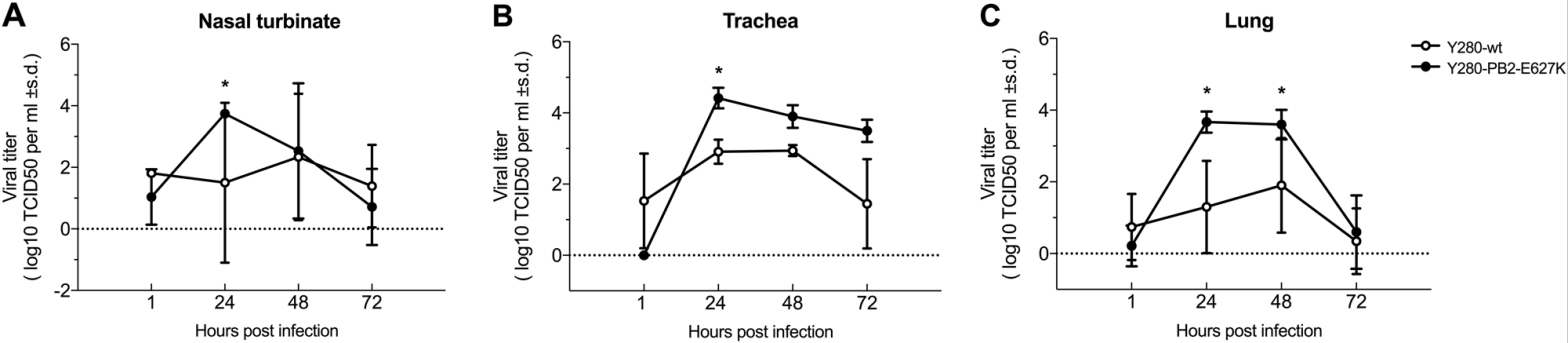
Viral replication of H9N2 viruses from ex vivo cultures of the tree shrew respiratory tract. Tissues isolated from different sites of the respiratory tract were infected with 10^6^ TCID_50_/mL of H9N2 viruses (Y280-wt or Y280-PB2-E627K) at 37 °C. Virus titers in ex vivo culture of tree shrew nasal turbinate (**a**), trachea (**b**), and lung (**c**) tissues (*n* = 3 per time point) were determined by TCID_50_ assays. Asterisks represent statistical significance compared to 1 hpi. *p < 0.05

We established an ex vivo culture model (nasal turbinate, trachea and lung) to compare the replication of the H9N2 virus and its PB2-E627K mutant. Under comparable infection conditions, we found that the Y280-PB2-E627K virus replicated more efficiently in the nasal turbinate, trachea, and lung tissues than the Y280-wt virus did (*p* < 0.05) (Fig. 6a–c).

**Fig. 7:**
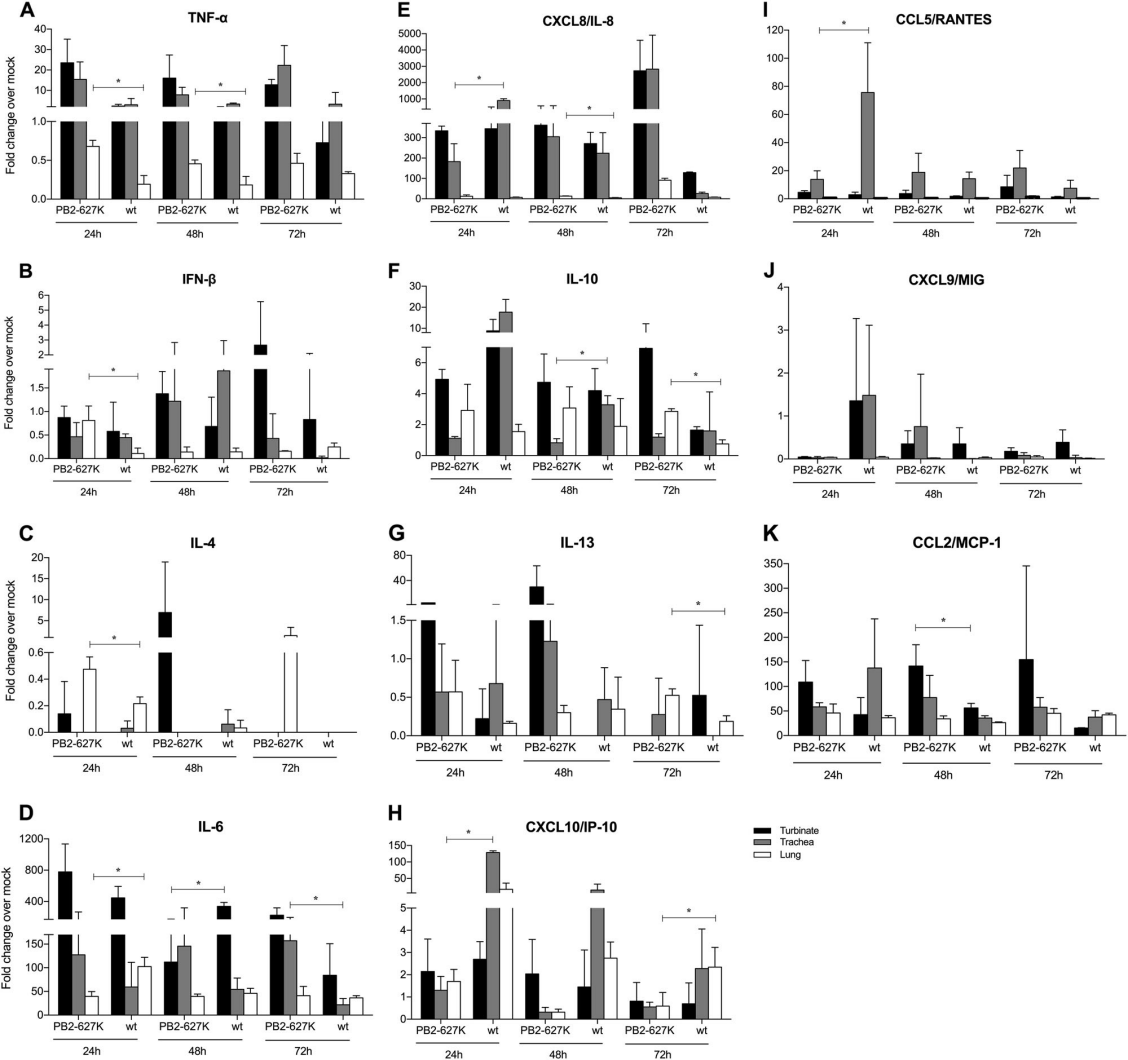
Cytokine mRNA expression from ex vivo cultures of the respiratory tract isolated from tree shrews. Tissues isolated from different parts of the respiratory tract were infected with 10^6^ TCID_50_/mL of H9N2 viruses (Y280-wt or Y280-PB2-E627K) at 37 °C. The mRNA expression levels of *TNF-α* (**a**), *IFN-β* (**b**),* IL-4* (**c**), *IL-6* (**d**), *CXCL8* (**e**), *IL-10* (**f**), *IL-13* (**g**), *CXCL10* (**h**), *CCL5* (**i**), *CXCL9* (**j**) and *CCL2* (**k**) in different ex vivo explants (*n* = 3 per time point) were measured by quantitative PCR. The expression of the target genes was standardized to the mRNA expression of the *GAPDH* gene and the expression in mock-infected tree shrews. *p < 0.05

The mRNA expression profiles of the selected cytokines were also measured from the ex vivo cultures after infection with H9N2 viruses (Fig. 7). The cytokine levels of *TNF-α* (24 h and 48 h), *IFN-β* (24 h), *IL-8* (48 h), *IL-10* (72 h), *IL-4* (24 h), and *IL-13* (72 h) were significantly higher in the ex vivo lung tissues infected with the Y280-PB2-E627K virus than in those infected with Y280-wt virus. In contrast, higher levels of *IL-8* (24 h), *RANTES* (24 h),* IP-10* (24 h), and *IL-10* (48 h) in the tracheas as well as *IL-6* (24 h) and *IP-10* (72 h) in the lungs were found in the Y280-w-infected group than in the Y280-PB2-E627K-infected group.

## Discussion

Our study aimed to characterize the susceptibility of tree shrews to avian H9N2 virus infection as part of an investigation to determine whether this experimental animal model is useful for studying the pathogenesis of avian influenza viruses in mammals. Unlike those with H5N1 or H7N9 viruses, patients with H9N2 infection mainly manifest mild upper respiratory symptoms, including fever, coryza, sore throat, and inflamed oropharynx, analogous to the effects of seasonal influenza viruses^2,22,23^. We found that infection with the wild-type avian H9N2 virus (Y280-wt) showed transient virus shedding without an obvious increase in body temperature or weight loss. However, our histological findings clearly identified evidence of infection, with inflammatory cells and focal edema observed in the submucosal layer of the nasal turbinates at the early stage of infection. Although one tree shrew showed no virus shedding after infection with the H9N2 wild-type virus, the high antibody titer against the H9N2/Y280 strain in the blood of this tree shrew suggested that there was asymptomatic infection.

Mutations in the *PB2* gene contribute to the pathogenicity of avian influenza virus in mammalian hosts^9–11^. Specifically, the PB2-E627K mutation is associated with an increase in the virulence and replication of the avian influenza virus in both in vitro and in vivo models. We also found that H9N2 virus with the E627K mutation (Y280-PB2-E627K) was shed by tree shrews in higher quantities and for a longer period than its wild-type control. The virus was disseminated more widely in the respiratory tract, and there was evidence of inflammation in the lower lung in some but not all tree shrews. Taken together, our results showed that the tree shrew is highly susceptible to avian influenza H9N2 virus infection.

Different experimental animal models have been used to simulate the outcomes of avian influenza virus infection in humans. Mice and ferrets are the two commonly used experimental animal models used for the in vivo study of avian influenza virus. Macaques have occasionally been used as an experimental model but are extremely costly; additionally, there are ethical issues with their use, and the outcomes of infection in macaques do not closely mimic those observed in humans. We and others have previously studied the pathogenesis of H9N2 virus infection and the contribution of the PB2-E627K mutation in mice^12–14^. It was consistently found that H9N2 virus with this mammalian adaptation mutation usually caused more severe disease and greater lethality and weight loss in mice than in humans. This more pronounced phenotype in mice may be due to the availability of α2,3-linked SA receptors in the upper respiratory tract of mice, which would facilitate lower lung infection even by intranasal inoculation of the virus^24^. The human and ferret respiratory tract predominantly has α2,6-linked SA receptors in the upper respiratory tract, with α2,3-linked SA receptors being present only in the lower respiratory tract. In our study, both ferrets and tree shrews supported the replication of the H9N2 virus, and this replication was further enhanced by the PB2-E627K mutation. However, the overall severity of infection in these two animal models remained mild, which is comparable to what is observed in humans. The ferret has been used as a model to assess the pathogenicity and transmissibility of the H9N2 virus because it has a distribution of α2,3 and α2,6 SA receptors that resembles the distribution seen in the human respiratory tract^25^. In our experiments, the wild-type H9N2/Y280 virus replicated efficiently in ferrets even without the PB2-E627K mutation, while the difference between the wild type and the PB2 mutant was more significant in tree shrews, supporting the previous finding that tree shrews are susceptible to avian H9N2 virus infection. Moreover, our newly established tree shrew ex vivo cultures demonstrated that the PB2-E627K mutation enhanced the early cycles of H9N2 virus replication in the nasal turbinate and trachea, in which the kinetics were consistent with our findings from in vivo infection. Interestingly, the PB2-E627K mutant clearly replicated more efficiently than the wild-type virus in the ex vivo study, while virus replication was observed only in the lungs of two tree shrews that were infected with the mutant. This result suggests that the PB2-E627K mutant has adapted by gaining the ability to replicate in the lower lung, but replication in this site may not occur in every infected individual and may be caused by other factors, which is similar to the situation in humans.

The induction of proinflammatory cytokine and chemokine expression has been shown to contribute to the pathogenesis of avian influenza virus infection^26^. We established a set of primers that can detect the mRNA level of 11 cytokines in the tree shrew. Elevated levels of cytokines were detected in the tissues isolated from the respiratory tract after infection with either the wild type or the PB2 mutant compared to the levels in the uninfected control, suggesting that infection with H9N2 virus can trigger innate immune responses in tree shrews. We found that the increase in proinflammatory cytokines in the in vivo experiments generally peaked at day 2 and decreased at day 4, matching the viral titers detected in nasal wash at the corresponding time point, which indicates a strong correlation between cytokine expression and virus replication in the upper respiratory tract. Transient cytokine expression during early infection was also reported in ferrets^27,28^. However, we did not find similar cytokine profiles between the in vivo and ex vivo experiments. This result is understandable, as the results from these two experiments were based on different infection conditions. While the former showed the regulation of cytokines in tree shrews under pathological conditions, the latter reflected the cytokine expression phenotype of wild-type H9N2 and its PB2 mutant in different tissues. Thus, the results from the two experimental settings provide different angles from which to characterize the pathogenesis of the virus.

In conclusion, our study further demonstrates the potential of the tree shrew as an additional experimental animal model for investigating the pathogenesis of influenza. The virus replication patterns and phenotype mimic what is observed with humans and ferrets. As an experimental model, the tree shrew is smaller in size, easier to handle, less aggressive and less expensive in terms of the cost per animal than ferrets (approximately 1/10 of the price). Importantly, tree shrews are evolutionarily closer to humans and primates than ferrets are^19,21^.

## Materials and methods

### Viruses and cells

H9N2 virus A/duck/Hong Kong/Y280/97 (Y280-wt) and its PB2-E627K mutant (Y280-PB2-E627K) were grown in 9- to 11-day-old specific-pathogen-free (SPF) chicken eggs. MDCK cells were maintained in Dulbecco’s modified Eagle’s medium supplemented with 10% fetal bovine serum at 37 ℃ with 5% CO_2_.

### In vivo experiments

Tree shrews (female/male, 100~130 g) and ferrets (female, 900~1000 g) were purchased from the Faculty of Life Science and Technology, Kunming University of Science and Technology, Kunming, China. The animals were confirmed as serologically negative for the H9N2 Y280-wt virus before use. The animals were first anesthetized via intraperitoneal injection of 3% pentobarbital sodium dissolved in PBS and inoculated intranasally with Y280-wt or Y280-PB2-E627K virus (day 0). Body temperature and body weight were recorded daily using subcutaneous transponders (IPTT-300; Bio Medic Data Systems Inc., Seaford, Delaware), starting 4 days prior to infection. At days 2, 4, and 6 postinfection, 0.25 mL (0.125 mL per nostril) of PBS was instilled into the nostrils of the infected animals and allowed to exit into a Petri dish. The fluid (nasal wash) was collected for virus titration using the TCID_50_ assay in MDCK cells. Seroconversion of the animals was tested by HI assays. All animals used in the experiments were humanely euthanized at day 21 postinfection. At days 2, 4, and 6 postinfection, four tree shrews were randomly selected for euthanasia, and nasal turbinate, throat, trachea, and lung tissues were obtained. The left and right nasal turbinates and lungs, as well as the left and right sides of the trachea, were set aside in equal portions. One portion was used for histopathological analysis and immunostaining with anti-influenza NP antibody. Another portion was homogenized with PBS supplemented with ofloxacin, amphotericin B, penicillin, and streptomycin followed by centrifugation at 13,000 × *g*, 4℃. The virus titer in the supernatant was determined by a TCID_50_ assay, and total RNA from the cell debris was extracted to measure the mRNA levels of the selected cytokines and chemokines.

### Ex vivo experiments

Ex vivo cultures of nasal turbinate, trachea, and lung tissue were carried out as previously described^29^. Briefly, the tissue blocks were first infected with 1 mL of 10^6^ TCID_50_ influenza viruses and washed with warm PBS five times after 2 h of incubation. The tissue blocks were then cultured with fresh medium (F-12K supplemented with l-glutamine, ofloxacin, amphotericin B, 100 U/mL penicillin, 100 μg/mL streptomycin, and 1.5 μg/mL TPCK) at 37 °C. At 1, 24, 48, and 72 hpi, the supernatant was collected for TCID_50_ assays. The tissue blocks were collected and homogenized with TRIzol (Invitrogen, USA). Total RNA was then extracted, after which the mRNA expression of the cytokines was measured using quantitative PCR.

### Quantification of mRNA

Total RNA was converted to cDNA using PrimeScriptTMRT Master Mix (Takara), and quantitative PCR was then performed with 2× H-TECH SYBR qPCR Mix according to the manufacturer’s protocols. The levels of cytokine mRNA expression were reported as fold changes compared to the results from mock-infected tree shrews. The primers were designed using Primer 5.0 and are listed in Supplementary Table 1.

### Histology and immunohistochemistry

The tissues isolated from the respiratory tract were first fixed with 10% neutral buffered formalin, embedded in paraffin, sectioned at 3 μm and stained with hematoxylin and eosin (H&E) as previously described^30^. Immunohistochemical staining of the influenza nucleoprotein in the tissues was performed as follows. Four-micron-thick sections were treated with 0.1% Pronase (Roche 10165921001, Mannheim, Germany) in Tris buffer (1 M, pH 7.2) at 37 °C for 15 min, transferred to 95 °C for 15 min, and then blocked with 3% H_2_O_2_ for 20 min, followed by treatment with a Streptavidin/Biotin Blocking Kit (Cat. No. SP-2002, Vector Labs, Burlingame, CA). After being blocked with 10% normal goat serum for 20 min, the sections were incubated with 1/10 HB65 (EVL anti-influenza NP, subtype A) primary antibody for 1 h at room temperature, followed by incubation with biotinylated goat anti-mouse secondary antibody (Cat. No. ab64257; Abcam, Cambridge, MA, USA) for 30 min at 37 °C. After incubation with the Vectastain ABC Kit (Cat. No. PK-6100, Vector Lab, Burlingame, CA), the sections were developed with the Vector NovaREDTM Substrate Kit (Cat. No. SK-4800, Vector Lab, Burlingame, CA).

### Statistics

Statistical analysis was performed using GraphPad Prism 7.0 software. Differences in viral titers and mRNA expression among groups were compared using an unpaired, parametric *t* test or the Mann−Whitney *U* test. *P* values < 0.05 were considered significant.

## Electronic supplementary material

Sup Fig 1

Sup Fig 2

Supplementary figure legends

Supplementary tables

## Data Availability

The datasets generated and/or analyzed during the current study are available from the corresponding author upon request.
