# Supplementary figures and images for: Tree shrew as a new animal model to study the pathogenesis of avian influenza (H9N2) virus infection
Source: Emerg Microbes Infect. 2018 Oct 10;7:166. doi: 10.1038/s41426-018-0167-1 (PMC6177411; doi:10.1038/s41426-018-0167-1)

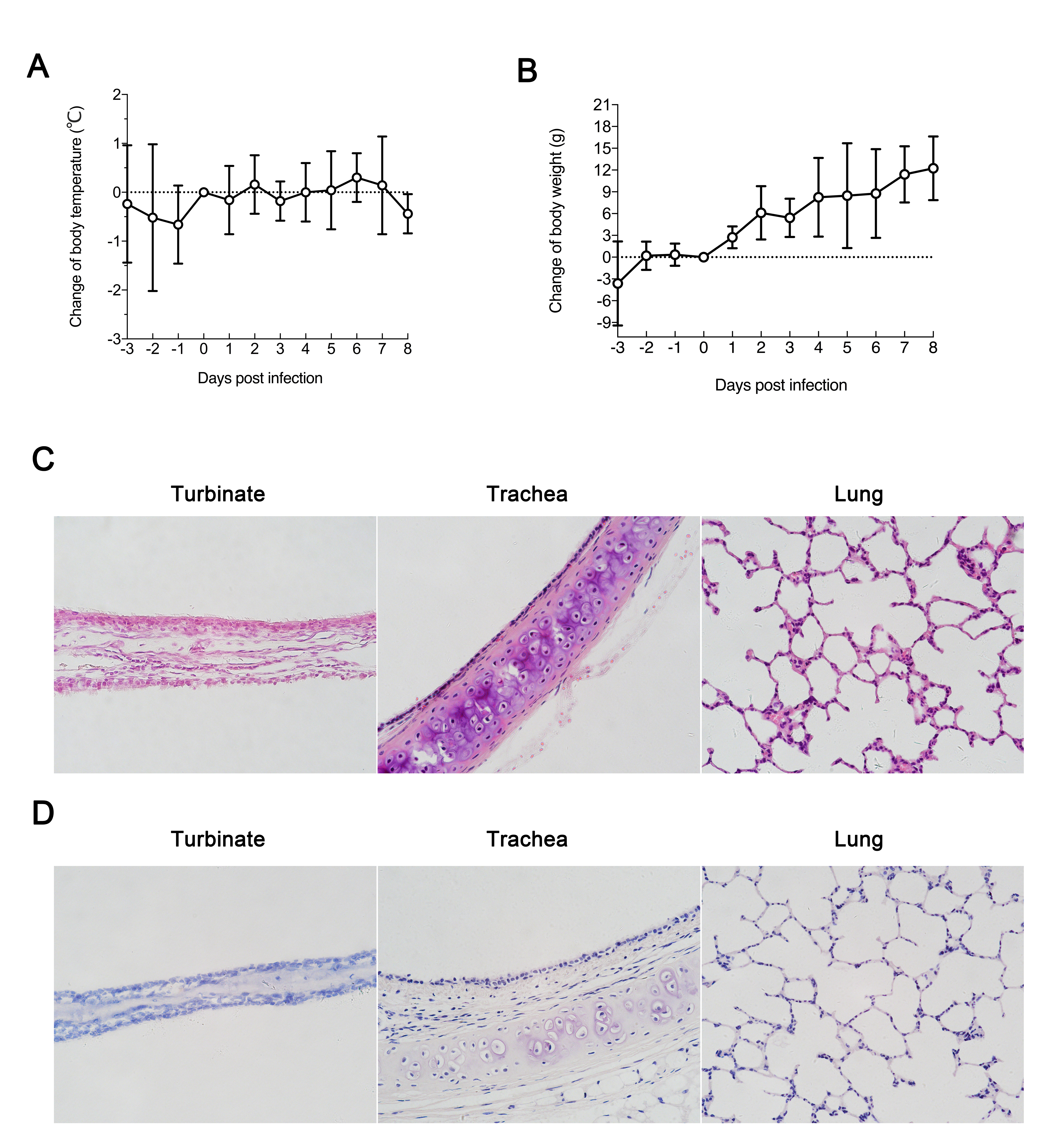

Supplement: Supplementary file 2 — Sup Fig 2 [file 41426_2018_167_MOESM2_ESM.jpg]
