# Supplementary material for: Tree shrew as a new animal model to study the pathogenesis of avian influenza (H9N2) virus infection
Source: Emerg Microbes Infect. 2018 Oct 10;7:166. doi: 10.1038/s41426-018-0167-1 (PMC6177411; doi:10.1038/s41426-018-0167-1)
Supplement: Supplementary file 3 — Supplementary figure legends [file 41426_2018_167_MOESM3_ESM.docx]

**Supplementary Figure 1. Histopathology of the respiratory epithelium of tree shrews infected with H9N2 viruses at 4 dpi.** Tree shrews (n=4 per group) were infected with 10^6^ TCID_50_ of H9N2 viruses (Y280-wt or Y280-PB2-E627K). Nasal turbinate (A, B), trachea (C, D), and lung (E, F) tissues were collected at 4 dpi, processed into paraffin sections and stained with H&E. Black arrows indicate infiltration of inflammatory cells, and white arrows indicate the presence of inclusion bodies. The images are shown at 400× magnification.

**Supplementary Figure 2. Body weight, body temperature, histopathology and immunohistochemical staining of mock-infected tree shrew.** Changes in body temperature (A) and body weight (B) were determined in tree shrews (n=3) that were mock infected and monitored for 12 days. Nasal turbinate, trachea, and lung tissues were processed into paraffin sections and stained with H&E (C) or influenza virus antigen by IHC (D).
