# Supplementary material for: Tree shrew as a new animal model to study the pathogenesis of avian influenza (H9N2) virus infection
Source: Emerg Microbes Infect. 2018 Oct 10;7:166. doi: 10.1038/s41426-018-0167-1 (PMC6177411; doi:10.1038/s41426-018-0167-1)
Supplement: Supplementary file 4 — Supplementary tables [file 41426_2018_167_MOESM4_ESM.docx]

Supp Table 1. Primer list for detection of cytokine mRNA levels

| Cytokine | Forward | | Reverse |
| --- | --- | --- | --- |
| TNF-α | 5′- GGAACAGTCACTCAAGAC-3′ | 5′- CATGGAACAAATGTGAAAGA-3′ | |
| IFN-β | 5′- GTTCCTACAGCAGTTGGG-3′ | 5′- TAGCGTTCTCTTTCTGGAA-3′ | |
| IL-4 | 5′-TTGCCGCATCCAAGAACACA-3′ | 5′-TGAGTCCTCTCAGGAGTCGGGC-3′ | |
| IL-6 | 5′- CAGTCCAGTTGCCTTCTC-3′ | 5′- GTCACATGCCTCTTGTTTC-3′ | |
| CXCL8/IL-8 | 5′-CACATTCGACGCCTTTTCACCC-3′ | 5′-TTTCCTTGGGATCCAGGCAGA-3′ | |
| IL-10 | 5′- GTGAGAACAAGAGCAAGG-3′ | 5′- GGCTTTGTAGACACCTTTC-3′ | |
| IL-13 | 5′-TGTCACCCAGAATCAAAAGGCG-3′ | 5′-TTGGTCAGGGACTCCAGGGCTA-3′ | |
| CXCL10/IP-10 | 5′- TGCCATTCTCCTTTTCTG-3′ | 5′- CGTACAGTTCGAGAGAGA-3′ | |
| CCL5/RANTES | 5′- GCCTACATCTCCCGCCCACT-3′ | 5′- TATTCCCGAACCCATTTCTT -3′ | |
| CXCL9/MIG | 5′-AAACAATACGCCCCAAGCCCT-3′ | 5′-TTCTTCACATTTGCTGAATCTGGG-3′ | |
| CCL2/MCP-1 | 5′-TGAACACCAACTGTCCTAAAGAAGC-3′ | 5′-CACTTCTGCTTGGGGTCAGCA-3′ | |
| GAPDH | 5′- TCGGAGTAAACGGATTTG-3′ | 5′- CCATGTAGTTCAGGTCAA-3′ | |
